# A Rare Case of Nasopalatine Duct Cyst Diagnosed 7 Years After Secondary Bone Grafting in a Patient With Cleft Lip and Palate

**DOI:** 10.1155/crid/5520791

**Published:** 2025-08-02

**Authors:** Kazuyuki Yusa, Satoshi Kasuya, Nobuyuki Sasahara, Tomoharu Hemmi, Shigeo Ishikawa

**Affiliations:** Department of Dentistry, Oral and Maxillofacial-Plastic and Reconstructive Surgery Faculty of Medicine, Yamagata University, Yamagata, Japan

**Keywords:** cleft lip and palate, nasopalatine duct cyst, secondary autogenous particulate cancellous bone and marrow grafting

## Abstract

Secondary autogenous particulate cancellous bone and marrow grafting (SBG) is a reliable and established technique for patients with alveolar cleft. Some reports have described complications after SBG, but cyst formation in alveolar bone where SBG has been performed appears to be extremely rare. Herein, we report a rare case of a cyst forming at a grafted site 5 years after SBG. A 15-year-old girl was referred to our clinic with a chief complaint of swelling in the right maxilla. The patient had a history of multiple surgeries for cleft lip and palate. Panoramic radiography and computed tomography (CT) revealed a radiolucency in the right maxilla, at the site where SBG had been performed. Enucleation of the lesion and bone graft using particulate cancellous bone and marrow were performed. The histological diagnosis was a nasopalatine duct cyst. Postoperatively, no signs of recurrence have been observed. Some reports have suggested that damage to the incisive canal can lead to the formation of nasopalatine duct cysts. SBG thus needs to be performed with care, as invasion of the incisive canal during the procedure may result in cyst formation and the need for additional surgery.

## 1. Introduction

Secondary autogenous particulate cancellous bone and marrow grafting (SBG) is a reliable and established technique for patients with alveolar cleft [1]. Postgraft bone resorption due to infection, wound dehiscence, and inadequate blood flow has been cited as common posttreatment problems. In addition, cysts such as nasolabial cysts have been reported to form in the soft tissue surrounding the alveolar cleft [2, 3]. On the other hand, cyst formation in alveolar bone where SBG has been performed appears to be extremely rare [4–6]. Herein, we report a rare case of a cyst forming at the grafted site 5 years after SBG.

## 2. Case Presentation

**Figure 1:**
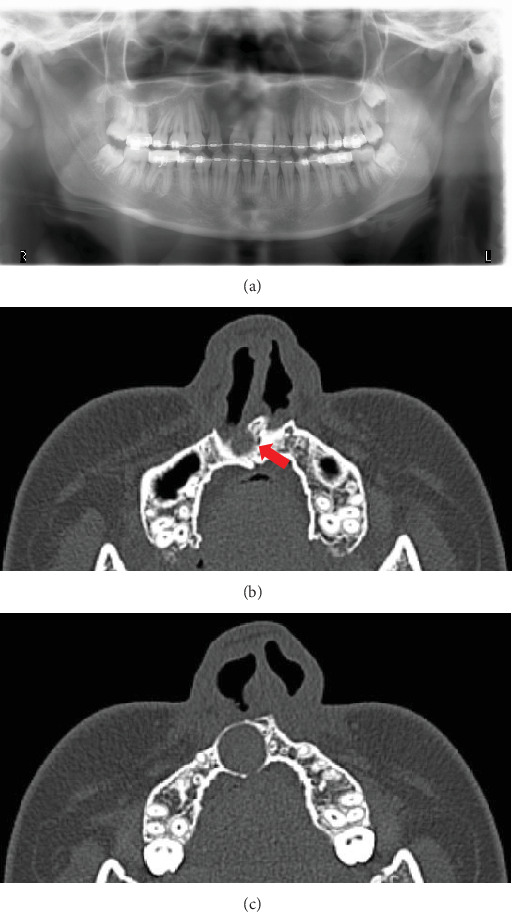
(a) Preoperative panoramic radiography and (b,c) CT. Continuity of the lesion with the incisive canal is confirmed (b, arrow).

**Figure 2:**
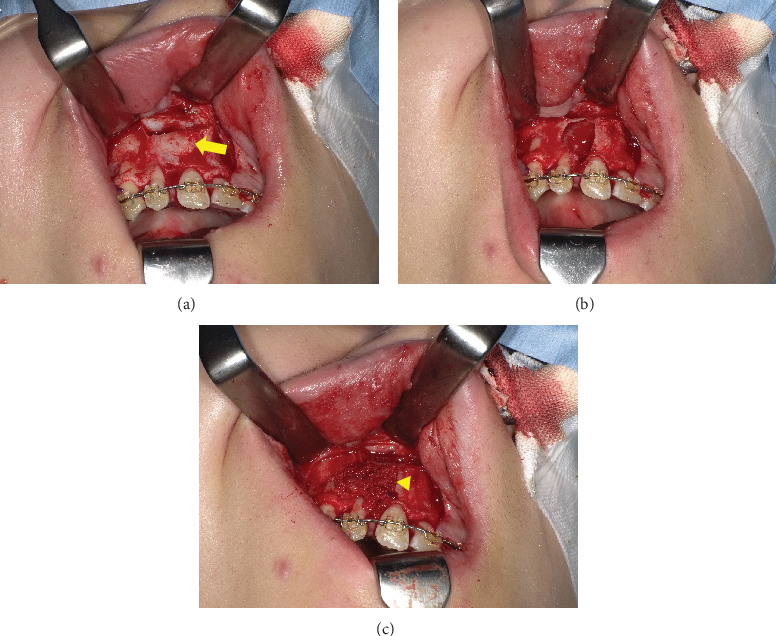
Intraoperative findings. The (a) cyst is covered with cortical bone (arrow) and (b) the cystic cavity is filled with (c) particulate cancellous bone and marrow (arrowhead).

**Figure 3:**
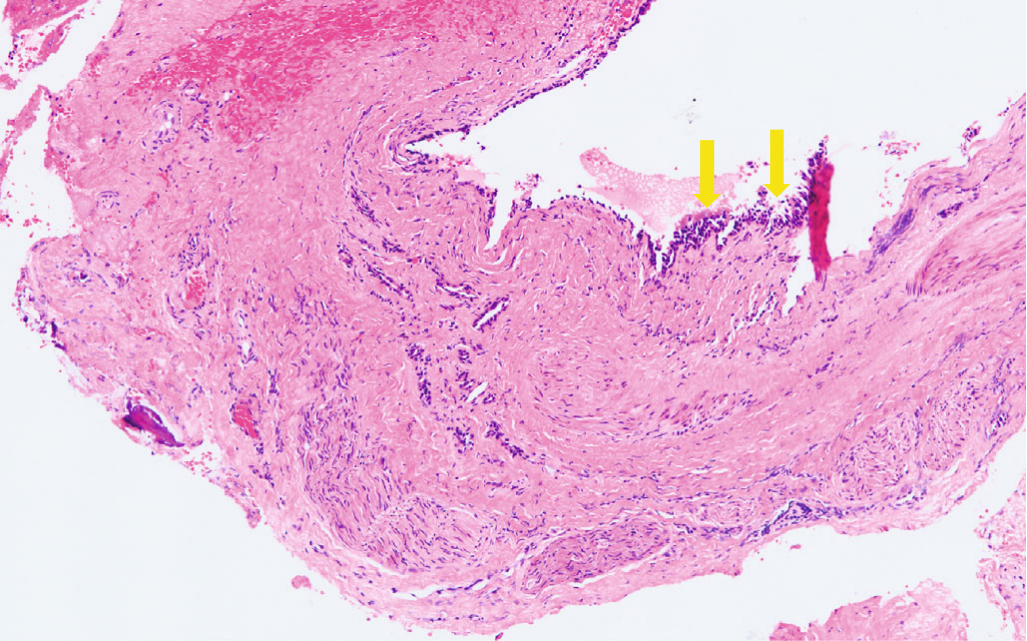
Micrographs of hematoxylin-eosin stained section of the cyst (100× magnification). The cyst is lined with pseudostratified ciliated epithelium (arrows).

A 15-year-old girl was referred to our clinic by her family orthodontic clinic with a chief complaint of swelling in the right maxilla. She was born with complete right cleft lip and palate. She underwent cheiloplasty at 3 months old and palatoplasty by double opposing Z-plasty at 18 months old. A second lip revision was performed at 7 years 8 months old, and SBG was performed to treat alveolar bone defect at 8 years 10 months old. These surgeries had been performed by the plastic surgery department of a nearby hospital, and swelling of the right palatal region was observed several times. The patient came to our department for close examination and a request for treatment, including future orthognathic surgery. Intraorally, no marked swelling was observed on the first visit to our hospital. Panoramic radiography (Figure 1a) and computed tomography (CT) (Figure 1b,c) revealed a well-defined, unilocular radiolucency approximately 15 mm in diameter in the right maxilla, at the site where SBG had been performed about 7 years earlier. A nasopalatine duct cyst was suspected as the preoperative diagnosis, although such cases are extremely rare. This was based on the continuity observed between the cyst and the nasopalatine duct on preoperative CT, as well as the fact that the surrounding teeth appeared intact, with no evidence of root resorption. Since orthognathic surgery was scheduled for a few years later, bone graft and enucleation of the lesion were performed to avoid residual bone defects that could interfere with orthognathic surgery. Particulate cancellous bone and marrow were harvested from the left posterior ilium, following the method described in previous reports [7, 8] (Figures 2a, 2b, and 2c). Histological examination of the cyst showed pseudostratified ciliated epithelium with a relatively large peripheral nerve and ductal epithelium, resulting in a histological diagnosis of a nasopalatine duct cyst (Figure 3).

**Figure 4:**
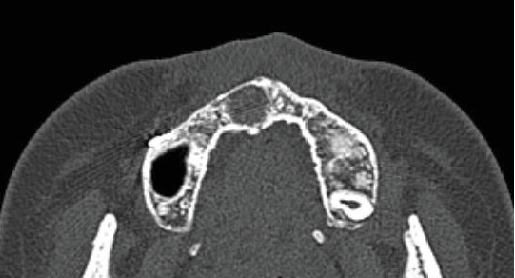
CT obtained 1 year after surgery shows no signs of recurrence.

Two years after this surgery, the scheduled orthognathic surgery was performed. The patient is currently under observation, with no sign of recurrence and good progress (Figure 4).

## 3. Discussion

Nasopalatine duct cysts develop from embryogenic remnants of the nasopalatine duct, a communication between the nasal cavity and the anterior maxilla in the developing fetus [9]. Nasopalatine duct cysts are considered a relatively rare condition that comprises less than 5% of jaw bone cysts [10]. At present, the detailed etiology of cyst formation in the nasopalatine duct cyst is unknown. However, Brode and Araiche reported that infection or trauma may trigger cyst formation [11]. Local infection or trauma is considered to induce irritation of epithelial cells, which may subsequently lead to cyst development [12]. The pathology has been considered to be associated with trauma, but several cases of nasopalatine duct cysts associated with implant placement have been reported in recent years [13–15]. These findings suggest that damage to the incisive canal at the time of dental implant placement can cause cyst formation, consistent with the hypothesis of cyst formation associated with trauma. In the present case, the patient had undergone cheiloplasty, palatoplasty, and SBG, so presumably, the incisive canal was invaded during SBG, leading to cyst formation. Based on the above history and histopathological findings, a nasopalatine duct cyst was diagnosed in this case.

Various cases of nasopalatine duct cyst at implant sites have been reported, including both cases in which the implant was preserved and only the cyst was removed [15], and cases in which the implant and cyst were completely removed [13, 14]. Casado et al. reported that cystectomy including implant removal may be necessary in some cases, taking into account the development of secondary osteomyelitis [13]. On the other hand, treatment for cysts in the jawbone, including nasopalatine duct cysts, after SBG has not been discussed due to the extreme rarity of this pathology. Possible treatment strategies include removal of the cystic lesion with subsequent closure of the wound, or filling of the cystic cavity with autogenous bone or alloplastic bone grafting material. The patient in this report was prepared for orthognathic surgery, with bone grafting into the cystic cavity to ensure optimal surgical technique and postoperative stability. As a result, the orthognathic surgery was successfully completed without complications, and the patient is currently doing very well.

In conclusion, we have reported a case of a nasopalatine duct cyst in the grafted bone after SBG, and careful performance of SBG is important, considering that invasion of the incisive canal during SBG may result in cyst formation and a need for additional surgery.

## Data Availability

The data that support the findings of this study are available on request from the corresponding author. The data are not publicly available due to privacy or ethical restrictions.
